# Advances in optogenetic studies of depressive-like behaviors and underlying neural circuit mechanisms

**DOI:** 10.3389/fpsyt.2022.950910

**Published:** 2022-09-08

**Authors:** Shanshan Lin, Yiwei Du, Yujie Xia, Yumeng Xie, Ling Xiao, Gaohua Wang

**Affiliations:** ^1^Department of Psychiatry, Renmin Hospital of Wuhan University, Wuhan, China; ^2^Institute of Neuropsychiatry, Renmin Hospital of Wuhan University, Wuhan, China

**Keywords:** anhedonia, appetite, depression, neural circuit, optogenetics, social avoidance

## Abstract

**Backgrounds:**

The neural circuit mechanisms underlying depression remain unclear. Recently optogenetics has gradually gained recognition as a novel technique to regulate the activity of neurons with light stimulation. Scientists are now transferring their focus to the function of brain regions and neural circuits in the pathogenic progress of depression. Deciphering the circuitry mechanism of depressive-like behaviors may help us better understand the symptomatology of depression. However, few studies have summarized current progress on optogenetic researches into the neural circuit mechanisms of depressive-like behaviors.

**Aims:**

This review aimed to introduce fundamental characteristics and methodologies of optogenetics, as well as how this technique achieves specific neuronal control with spatial and temporal accuracy. We mainly summarized recent progress in neural circuit discoveries in depressive-like behaviors using optogenetics and exhibited the potential of optogenetics as a tool to investigate the mechanism and possible optimization underlying antidepressant treatment such as ketamine and deep brain stimulation.

**Methods:**

A systematic review of the literature published in English mainly from 2010 to the present in databases was performed. The selected literature is then categorized and summarized according to their neural circuits and depressive-like behaviors.

**Conclusions:**

Many important discoveries have been made utilizing optogenetics. These findings support optogenetics as a powerful and potential tool for studying depression. And our comprehension to the etiology of depression and other psychiatric disorders will also be more thorough with this rapidly developing technique in the near future.

## Introduction

Depression is a kind of psychiatric disorder with high prevalence and suicidal incidence. According to the World Health Organization, depression accounts for 10% of worldwide disability caused by non-infectious diseases and turns out to be one of the leading reasons for disability (1, 2). Depression has a complicated etiology that has yet to be fully understood. Current opinions suggest that neuroinflammation, neural plasticity, gut-brain axis and other factors are involved in the process (3–5). Besides, the effects of therapies nowadays still have limitations for major depressive disorder (MDD). Previous research has indicated that the alteration or dysfunction of some brain regions is relevant to the occurrence of depression (6). Thus, insights into the role that neural circuits play in depression may lead us to a better understanding of the etiology, symptoms, and treatment of depression.

Optogenetics, which combines the advantages of genetics and optical methods, is a technology that allows the control of specific neurons in living animals while monitoring their effects on behavior and physiology (7). Scientists have paid much attention to optogenetics in psychiatric disorders, behavior and recognition since opsin was used to trigger neuron activity for the first time by Karl Deisseroth in 2005 (8). For instance, astrocytes play an important role in stress response and inducing depressive-like behavior *via* glucocorticoid receptors (9). Many studies have used optogenetics to precisely modulate astrocyte activation, investigating its effects in sleep, cognition and other behaviors (10–12). Moreover, optogenetics has also provided us with a more efficient method of learning the effects of neural circuits on psychiatric disease.

This review gives a brief overview of optogenetics as well as recent breakthroughs in this field. The latest progress in learning the mechanisms of depressive-like behaviors utilizing optogenetics will be presented in detail. However, studies on neural circuits and brain regions are numerous and complicated, only those studied thoroughly, such as the medial prefrontal cortex (mPFC), ventral tegmental area (VTA), and hippocampus are included in this review. Finally, the therapeutic potential of optogenetics in clinical practice like ketamine and deep brain stimulation (DBS) will be discussed.

## Optogenetics

Optogenetics has become a hot topic in neuroscience during the past decades. With this technology, the microbial opsin gene is sent and integrated, by virus vector or recombinase system, into specific neurons in regions of interest and expresses excitatory or inhibitory opsin. The light with certain wavelength and frequency is then delivered in optic fiber to activate or inhibit target neurons. Thus, we can observe the impact of different brain regions and neural circuits on behavior and psychiatric disorders (13). This innovative technology combines the advantages of both genetic and optical methods: (i) High temporal resolution. Immediate neuronal activity control can be achieved by adjusting light wavelength, frequency and intensity; (ii) High spatial resolution. Light-sensitive opsins expressed in targeted neurons would precisely regulate action potential generation and firing in these cells; (iii) Low damage. Optogenetic technology causes less damage to experimental animals compared with traditional methods like electrical stimulation (14). Here, we mainly introduce the three essential components in optogenetics.

### Opsin family

Opsin is a kind of light-sensitive ion channel/ion pump protein that is widely distributed in nature (15). Opsins are divided into two main families: microbial opsins (type I rhodopsin, ion transport proteins) and animal opsins (type II rhodopsin, G protein-coupled receptors, and melanopsin) (16). Microbial have two different types based on the effects: depolarizing microbial opsins (channelrhodopsins, ChR2) and hyperpolarizing microbial opsins (bacteriorhodopsins, BR and halorhodopsins, NpHR). Most optogenetic studies choose ChR2 for cellular activation (17). ChR2 is a non-specific cation channel that is activated by blue light and permits Na^+^ and Ca2^+^ to enter cells, depolarizing targeted cells (18). While BR (proton pump activated by green light) and NpHR (chloride pump activated by yellow light) will hyperpolarize neurocytes and inhibit firing after illumination (14).

The past decades have seen great progress in the opsin family to meet different needs in size, kinetic properties and wavelength sensitivity with the development of modern genomics. Faster-deactivating ChR variants, such as ChEAT, reduce extra spikes occurring in response to a single light pulse and evoke sustained spike trains up to at least 200 Hz (19). Another ChR variant ChEF is made by chimeras of the transmembrane domains of ChR1 and ChR2 combined with site-directed mutagenesis. ChEF exhibits a significantly lower inactivation rate during persistent light stimulation. Moreover, point mutation of Ile^170^ in ChEF to Val has yielded a different variant, named ChIEF, that accelerates the channel closure rate while retaining reduced inactivation (20). ChEAT, ChEF, and ChIEF all allow for more precise temporal control of depolarization and can elicit action potentials that better mimic natural kinetics.

On the contrary, step-function opsin (SFO) is a modified ChR2 variant that can be activated by blue light and turned off by green light. After being exposed to blue light, the SFO keeps opening and maintains the cell depolarized for about 10 min. This method reduces the damage to animals' brains caused by consecutive high-power laser pulses (21). SwiChR, a step-waveform inhibitory ChR, for example, does not hyperpolarize neurons but instead opens up the Cl^−^ channel stably and reversibly. SwiChR has slower kinetics than endogenous GABA_A_ receptors, hence it is more used for long reversible inhibition experiments (22, 23). Investigators have engineered a new type of SFO with a longer spontaneous deactivation time constant approaching 30 min. One of the advantages of having this stabilized SFO (SSFO) is to conduct behavioral protocols in the absence of tethered external light delivery devices. As a result, we can observe animals' activities without affecting their natural behavior (24).

Adjustments are also made in wavelength sensitivity. Red light has longer wavelength and consequently has better tissue penetration compared with blue light, making it easier to optically control deep brain regions. VChR1 is a red-shift channelrhodopsin from Volvox carteri that can drive spiking at 589 nm, with excitation maximum red-shifted ~70 nm compared with ChR2 (25). The red-activatable Channelrhodopsin (ReaChR), a modified VChR1 variant is then developed. ReaChR is optimally excited with light from 590 to 630 nm and offers improved membrane trafficking, higher photocurrents, and faster kinetics compared with VChR1 (26).

### Viral vector and recombinase system

As microbial opsins can only produce small photo-currents and neural circuit components are highly diverse, high opsin gene expression in living animal's nervous system, targeting defined cell types or projections and minimizing cytotoxicity are crucial for optogenetics (7). Today, genetic specificity in optogenetics is realized mainly *via* three strategies, viral vector, recombinase-expressing driver animal lines and anatomical targeting strategies (13). Opsin expression in specific cells is successfully achieved by injecting adeno-associated virus (AAV) vectors or lentivirus under the control of a cell type-specific promoter. Promoter fragments conferring cell type preference are linked to markers like calcium/calmodulin-dependent protein kinase II subunit-α (CaMKIIα), hypocretin, oxytocin, or D2 dopamine receptor (17). However, only sequences that are < ~4 kb, specific, and strong can be packed into viruses, limiting their use in a wide range of cell types.

Utilizing the recombinase system would obviate the limitation and allow for larger promoter fragments. Transgenic mice are injected with recombinase-dependent opsin-expressing viral vector or along with targeted viruses that drive recombinase expression to realize opsin expression in cells of interest (13). This system can provide higher targeted opsin expression selectively in recombinase-expressing cells in the injected brain regions compared with viral vectors (27). For example, using Cre driver lines to comprehensively and selectively label brain-wide connections by layer and projection neuron class, Harris et al. revealed that class-specific connections are organized in a shallow hierarchy within the mouse cortical thalami network (28).

### Light delivery and readout equipment

The last essential components in the optogenetic toolbox are light delivery and signal readout devices. In general, a typical device of light delivery contains external light resources [the most popular light source is laser or light-emitting diodes (LEDs)], controller equipment, optical fibers and probes. This set of devices could provide effective optogenetic stimulation that consists of light with sufficient intensity to reach the designated location and enough energy to activate the expression of opsin, triggering a cellular signaling cascade (16). But there are several obvious defects. The physical tethers restrict animals' natural activity and behavior unavoidably, which affects the application and repeatability of the experiments. Optical fiber implantation into certain brain regions by invasive surgical operation causes side effects like brain damage, infection and bleeding. Besides, heat accumulated during illumination also hurts brain cells. The appearance of wireless devices implemented with miniaturized circuitry and specialized antenna designs makes it possible for researchers to observe the behavior, physiology and pathology of animals in a natural condition (29). To reduce injury, microscale light-emitting diode (uLED) arrays and tapered optical fibers have been widely applied in recent years, which meanwhile improve the spatial accuracy of optogenetic stimulation (30, 31).

Whole-cell patch-clamp electrophysiology was developed by Erwin Neher and Bert Sakmann and used for measuring single-channel currents. Although it is the gold standard for monitoring synaptic input and spiking output, it is still difficult to link the resulting data stream to defined cell types *in vivo* during behavior. This limitation can be circumvented by the integration of patch-clamp techniques with projection-targeted optogenetics (13, 32). Another combination of optogenetics with fast readouts creates the possibility of closed-loop optogenetic interventions, in which photostimulation is guided by real-time readout of ongoing activity. It gives us a chance to begin to understand the casual relationship between neural activity and behavior. Scientists can dynamically control neuronal activity patterns in awake animals and observe the function of neural circuits (16).

## Search strategy, study selection, and data extraction

**Figure 1:**
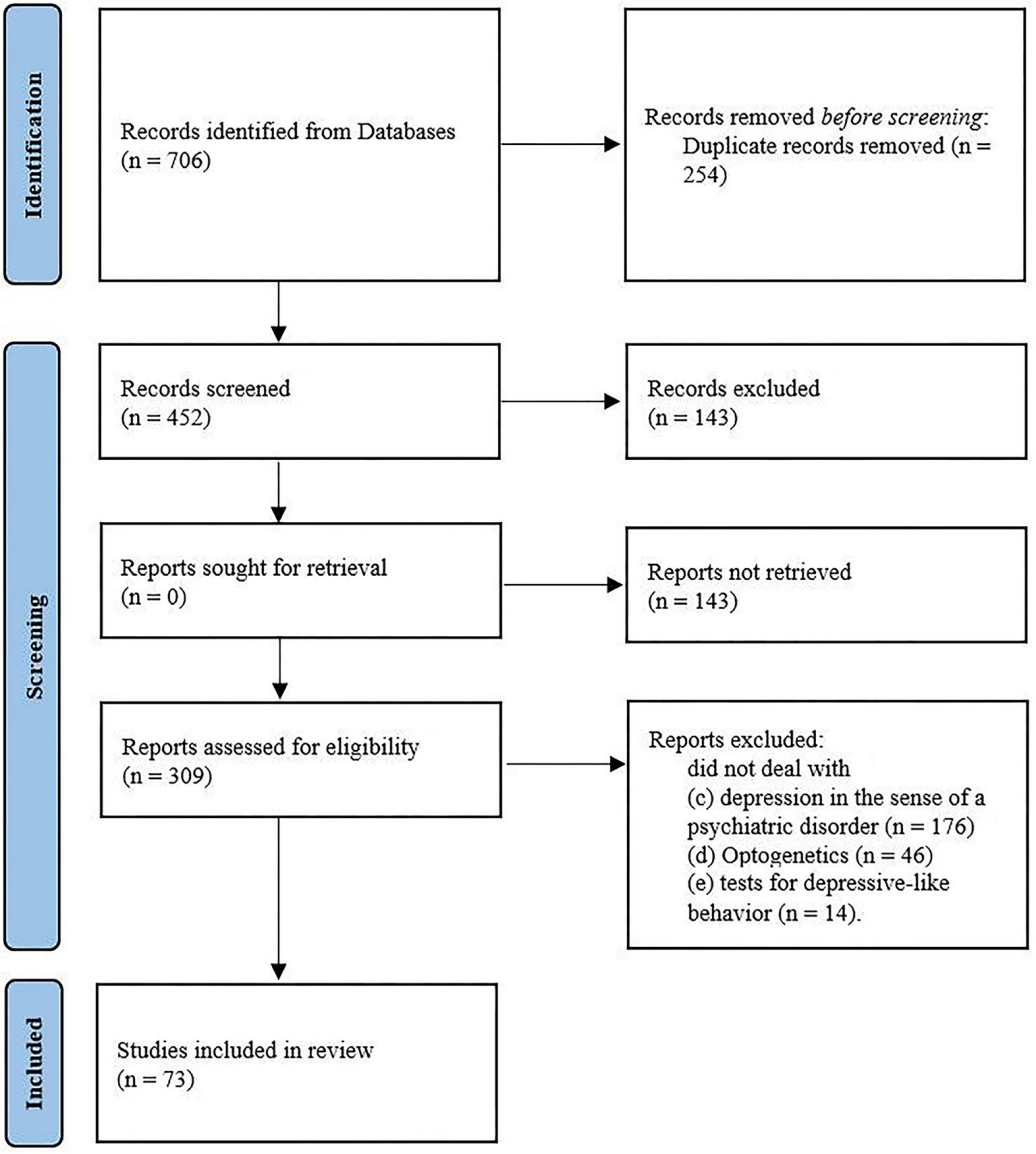
Flow chart of study selection by systematic search process according to the PRISMA group (2020).

Candidate studies were identified *via* PubMed and Web of Science by using the following search strategy:

The applied key terms were [(“depression” OR “depressive-like behavior” OR “anhedonia” OR “appetite” OR “social withdrawal” OR “social avoidance”) AND (“optogenetic”) AND (“mice” OR “rats”)] searched for in the topic and/or and other fields in the database. The search was restricted to articles written in English and published between database inception and April 26, 2022. In sum, 706 records were extracted. The set was refined by removing (a) duplicate entries (*n* = 254). Titles and abstracts were then screened for relevance, removing (b) reviews, meta-analysis, case studies, study protocols and books (*n* = 143). The full text of the remaining 309 papers was then assessed for eligibility. Subsequently excluding studies that did not deal with (c) depression in the sense of a psychiatric disorder (*n*= 176), (d) optogenetics (*n* = 46), (e) tests for depressive-like behavior (*n*=14). Figure 1 shows the sample development throughout the selection process. The study selection and eligibility screening were conducted according to the preferred reporting items for systematic reviews and meta-analyses (PRISMA) guidelines (33).

## Optogenetic findings in depressive-like behaviors

Major depressive disorder (MDD) is a multifaceted illness. MDD consists of a wide range of emotional, cognitive and behavioral symptoms, including core symptoms (anhedonia and depressed mood) as well as other symptoms (appetite changes, social withdrawal, sleep disturbance, worthlessness or self-guilt, and suicidal ideation). We have confirmed that the dysfunctional circuits underlying these symptoms include emotional, cognitive, perceptive and reward systems (34). And symptoms such as anhedonia have been well imitated in animal experiments. However, the existing problem is that symptoms like the feeling of worthless, self-guilt and suicidal ideation cannot be measured quantitively by behavioral tests like sucrose preference test (SPT) or tail suspension test (TST). Therefore, we only discuss publications that deal with anhedonia, appetite change and social avoidance/withdrawal in this section.

### Anhedonia

Anhedonia, as one of the core symptoms of depression, is defined as the reduced ability to feel pleasure in normally pleasurable situations (35). Anhedonia is a negative prognostic factor in the therapeutic outcomes of depressed patients (36, 37). Besides improvement of anhedonia significantly correlates with better overall functioning in depressed patients and is a strong prediction for psychiatrical functioning improvement (38, 39), which emphasizes its critical role in depression.

The clinical phenotype anhedonia is now considered to reflect dysfunctional processing of the reward network. Large numbers of reviews have focused on reward circuits in animals and humans (40, 41). The most consistently described reward network is the dopaminergic mesolimbic pathways originating from the VTA and spreading into the nucleus accumbens (NAc) located in the ventral striatum (VS), bed nucleus of the stria terminalis, amygdala, and hippocampus (42).

An animal study investigated the role of VTA in the reward network and found that optical activation of the VTA leads to dopamine release in the NAc and the establishment of conditioned place preference (CPP) (43). With eNpHR3.0 inhibiting DA neurons in VTA, mice show a significant decrease in SPT and also in forced swim test (FST) and TST. Furthermore, phasic illumination of ChR2-VTA DA neurons can reduce immobility time in TST and reverse the anhedonic effect in mice undergoing time-intensive chronic mild stress (CMS) paradigm (44). Another research shows a contradictory result in SPT after phasic firing of VTA DA neurons in mice undergoing subthreshold social defeat paradigm. Phasic photostimulation of VTA DA neurons also induces susceptibility to social defeat stress in mice (45). One possible reason for the bidirectional effects of VTA DA neurons may lie in the different stimulus paradigms. More detailed studies are required to explain the potential mechanisms because of the heterogeneity. Optogenetic activation of DA neurons from VTA projecting to NAc core and shell and infralimbic (IL)-mPFC induces positive reinforcement behavior and intracranial self-stimulation (46). A newly identified subtype of VTA DA neurons molecularly defined by NeuroD6 (NEX1M) also induces significant place preference behavior after illumination *in vivo* (47). And optical stimulation of D1 neurons in the downstream area NAc enhances CPP for cocaine in mice while activating D2 neurons attenuates this preference (48). Optical stimulation of lateral hypothalamic (LH) orexin/dynorphin inputs in the VTA potentiates mesolimbic dopamine neurotransmission in the NAc core and produces real-time place preference (49). The communication between neural populations within the LH and DA neurons in VTA and NAc is also demonstrated in earlier studies (50–52).

To our knowledge, 60% of efferent projections of the VTA are dopaminergic, and they also contain glutamatergic and GABAergic neurons (53). van Zessen et al. found optical activation of VTA GABA projections to the NAc did not disrupt reward consumption, which is contradictory to the decreased reward consummatory licking with VTA GABA activated during the 5 s period following reward deliver. These findings may imply that activation of VTA GABAergic projections in NAc alone was not sufficient to suppress reward consumption. Interestingly, activation of VTA GABA neurons simultaneously reduced the excitability and activity of VTA DA neurons (54). Photostimulation of vesicular glutamate transporter 2 (VGLUT2) neurons in VTA co-releases GABA/glutamate in projecting regions NAc, VP, and LHb. Nevertheless, the overall effect of the stimulation is serving as a potent reinforcer on positive behavioral tasks (55). Optogenetic activation of a dorsal raphe (DR)-originating pathway reinforces instrumental behavior and establishes conditioned place preferences. Notably, this pathway consists of VGLUT3-containing neurons projecting to VTA dopamine neurons, indicating that the DR-VLGUT3 pathway to VTA utilizes glutamate as a neurotransmitter and is a substrate linking DR to VTA dopaminergic neurons (56). All the discoveries above confirm that GABAergic and glutamatergic neurons engage in reward circuits, and reflect the complicated inner connections between dopaminergic, GABAergic and glutamatergic neurons.

The complexity of VTA in the process of anhedonia outlines its significance as an integral hub for corticolimbic circuitry. Besides, other evidence shows that mPFC, lateral habenula and other regions also contribute to anhedonia (57–59). Undoubtedly, anhedonia-related neural circuits act as critical factors in depression due to their core status in the symptomatology of depression. The function of VTA and other neural circuits in the etiology of depression will be further discussed in the next section.

### Appetite change

Appetite change, often accompanied with altered dietary habits and eating behavior, is a common symptom in patients with MDD. About 48% of adult depressed patients are reported to exhibit depression-related appetite decreases, while 35% exhibit depression-related increases in appetite (52, 60).

The hypothalamus has been proved to be the center of appetite regulation and optogenetics gives us a chance to further study how the hypothalamus and other neural circuits regulate appetite. The agouti-related protein (AgRP) neurons are a population of neurons regulating appetite in the arcuate nucleus of hypothalamus (ARH) (61). Another region, the parabrachial nucleus (PBN) also contains subpopulations of neurons that modulate appetite suppression (62). A previous study has shown that ablation of AgRP neurons hyperactivates PBN, causing starvation in adult mice (63). Glutamatergic neurons in the nucleus tractus solitarius (NTS) is then found to be the source of the excitatory inputs to the PBN. Activation of this pathway inhibits feeding, while genetic inactivation of glutamatergic signaling by the NTS onto NMDA-type glutamate receptors in the PBN prevents starvation. Optically suppressing glutamatergic output of the PBN reinstates normal appetite after AgRP neuron ablation (64). Calcitonin gene-related peptide (CGRP)-expressing neuron is another population identified in the outer external lateral subdivision of the PBN which projects to the laterocapsular division of the central nucleus of the amygdala (CeAlc). Optogenetic activation of PBelo CGRP neurons projecting to the CeAlc suppresses appetite. In contrast, photoinhibition of these neurons increases food intake and prevents starvation in AgRP neuron ablated mice (62). Moreover, stimulating AgRP neurons with optogenetic methods can reverse the appetite suppressing effects during chemogenetic-mediated stimulation of CGRP neurons. This result demonstrates that AgRP neurons are sufficient to decrease activity in anorexigenic PBN CGRP neurons, thereby increasing food intake (65).

The pro-opiomelanocortin (POMC)-expressing neurons existing in the ARH also participate in appetite regulation (61). Jeong et al. demonstrated that ARH POMC neurons express capsaicin-sensitive transient receptor potential vanilloid 1 receptor (TRPV1)-like receptor. Selective optogenetic stimulation of TRPV1-like receptor-expressing POMC neurons at 10 Hz for 1 h significantly decreases food intake (66). Besides, optogenetically stimulating ChR2-expressing ARH^POMC^ reduces short-term food intake *via* ARH^POMC^→ medial amygdala (MeA) pathway (67). From an electrophysiological standpoint, stimulation of AgRP neurons triggers a long-term depression of spontaneous excitatory postsynaptic current (sEPSC) in downstream POMC neurons, which can be enhanced by food deprivation (68). One possible mechanism that underlies the regulatory mechanism of POMC neurons is the rapamycin complex 1 (mTORC1) signaling. Nicolas et al. found inhibiting mTORC1 increases food intake by decreasing POMC neuron activity. They further identified two different POMC cells: POMC/GABAergic neurons activated by rapamycin and POMC/glutamatergic neurons inhibited by rapamycin. Acute inhibition of mTORC1 activity after optogenetic activation of POMC neurons reduced POMC/GABAergic transmission and increased POMC/glutamatergic transmission at the same time. This study indicates the heterogeneity of POMC function relies on mTORC1 signaling, which makes POMC neurons regulate appetite bidirectionally (69).

Apart from AgRP and POMC neurons, other populations in the hypothalamus also contribute to appetite regulation, including a subset of nucleus of the solitary tract neurons containing cholecystokinin (CCK^NTS^) (70), glutamatergic neurons projecting to PVH in the medial septal complex (MSc) (71), tachykinin-1 expressing neurons (PSTN^Tac1^) in the parasubthalamic nucleus (72), steroidogenic factor 1 (SF1) neurons in the ventromedial hypothalamus (73), and melanin-concentrating hormone-expressing neurons (74). In addition, progress has also been made in some known appetite-regulating factors like neuropeptide Y (NPY) and orexin with optogenetics (75, 76). Many other brain regions are also discovered to engage in appetite change in both clinical and basic experiments (77–80). In conclusion, it is enlightening for us to study appetite change in a hypothalamus-centered direction. Furthermore, we should consider this pathological behavior together with depression in the hope that we better understand this issue in depressed patients.

### Social avoidance/social withdrawal

Patients with MDD usually have trouble with normal social behavior, for instance, they may lose interest or pleasure in social activities and interactions, even developing to social withdrawal (or social avoidance). Humans are a gregarious species and interpersonal communication is an indispensable part of people's daily routine, so it is crucial to understand how depression affects their social behavior (81).

Social defeat is one of the most popular paradigms to model stress-induced depression, characterized by the similarities in social behaviors between mice and humans. The test mouse is placed with an aggressive mouse for 10 min every day and will be attacked by the resident one. In the next 10 days, the test mouse is forced to live in visual, olfactive and auditive but not physical contact with the aggressor. After this protocol, the animals display typical depressive-like behaviors, such as social avoidance and increased immobility time in TST and FST (34, 82).

Researchers have revealed how mPFC and its circuits affect social behavior. Earlier experiments showed optogenetic stimulation of glutamatergic or GABAergic neurons in mPFC in mice exposed to social defeat stress all exhibited anti-depressive responses including less social avoidance and improvement in SPT (83). Furthermore, optical activation in left PL- mPFC can reverse social avoidance, while inhibition leads to social avoidance. However, the same effect does not appear when regulating the right PL-mPFC, indicating stress-induced social avoidance is developed due to the dysfunction of the left mPFC (84). At the circuit level, a circuit involving mPFC and basolateral amygdala (BLA) is identified. Activation of descending projections from mPFC→ BLA abolishes social preference and produces behavioral avoidance (85). Interstingly, sub-circuits of mPFC→ BLA pathway exhibit opposite effects. Chemogenetic activation of PL-BLA or inhibition of IL-BLA circuitry impair social behavior in mice (85). Afferent projections to mPFC also shape social avoidance behavior. Inhibiting VTA-NAc and VTA-mPFC projections with optogenetics, scientists have induced resilience and susceptibility to social defeat stress, respectively (45).

The dorsal raphe nucleus (DRN) also contributes to social disorders. Sakurai developed a technology, Capturing Activated Neural Ensembles or CANE. Optogenetic silencing of CANE-captured social fear neurons is sufficient to result in reduced social avoidance (86). Challis et al. combined optogenetics with cFOS mapping and slice electrophysiology and revealed that photoinhibition of the projection from vmPFC to GABAergic neurons in the DRN could prevent the acquisition of social avoidance behavior (87). Another experiment confirmed that optogenetic activation of DA neurons in DRN could increase social preference as well (88). Projections originating from mPFC to different parts of thalamus are also demonstrated to regulate certain aspects of social behavior (89–91).

## Optogenetic findings in dysfunctional neural circuits

**Figure 2:**
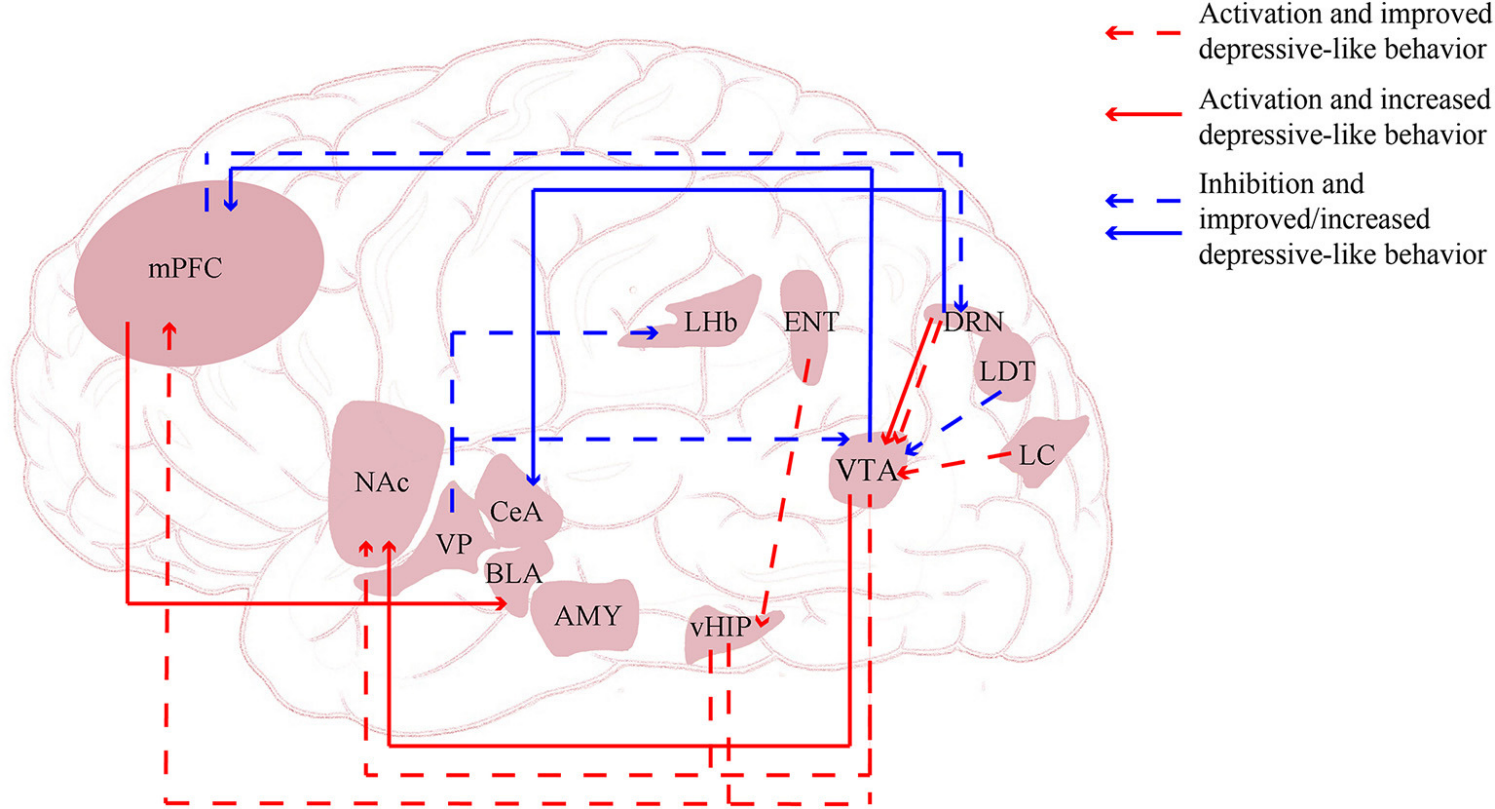
Neural circuits regulated *via* optogenetics and chegenetics. AMY, amygdala; BLA, basolateral amygdala; CeA, central nucleus of the amygdala; DRN, dorsal raphe nucleus; ENT, entorhinal cortex; LC, locus coeruleus; LDT, lateral dorsal tegmentum; LHb, lateral habenula; mPFC, medial prefrontal cortex; NAc, nucleus accumbens; vHIP, ventral hippocampus; VP, ventral pallidum.

The mechanisms underlying the pathophysiology and treatment of depression and stress-related disorders remain unclear, but studies in depressed patients and animal models are beginning to yield promising insights. Brain imaging studies have demonstrated altered connectivity and network function in the brains of depressed patients. Thus, an increasing number of studies are focusing on the function and dysfunction of neural circuits and networks in depression (see Figure 2).

### Medial prefrontal cortex

An imaging study of depression revealed robust outcomes regarding prefrontal cortex function, which primarily proved its effect on depression (92). Subsequent experiments have made further explorations. Susceptible mice subjected to chronic social defeat stress (CSDS) show fully restored social interaction times in behavioral tests after *in vivo* illumination of mPFC (83). Different Paradigm like maternal separation (MS) protocol can affect depression-like phenotypes in mice *via* mPFC activity (93). Son et al. also found transient anti-depressive effects after optogenetic stimulation of glutamatergic neurons in the mPFC (94). Sirtuin 1 (SIRT1) is a kind of NAD^+^-dependent deacetylase in forebrain excitatory neurons. Selective deletion of these neurons causes depression-like phenotypes only in male mice, while AAV-Cre-mediated STIR1 knockdown in the mPFC of male mice induced sex-specific depressive-like behaviors (95).

The connections between mPFC with other brain regions also attract great interest (96). Studies on the neurobiological basis of mPFC relevant alterations have focused on glutamatergic, 5-HT neurons as well as inhibitory GABA interneurons (97). The DRN is a crucial node of the widespread network implicated in emotional regulation. Selective optogenetic activation of mPFC cells projecting to the DRN induces a rapid and robust increase in kick frequency during FST, but does not affect non-specific locomotor activity in OFT (98). The mPFC provides a direct monosynaptic, glutamatergic drive to both DRN 5-HT and GABA neurons. Activation of cannabinol (CB) receptors differentially modulates the mPFC inputs onto these cell types in the DRN, finally leading to a robust increase in the synaptic excitatory/inhibitory ratio in DRN 5-HT neurons (99). Srejic et al. inhibited ChR2-5-HT neurons in the DRN with blue light stimulation. Serotonin clearance was significantly faster after 5 min high-frequency stimulation, indicating less release or more effective 5-HT reuptake (100). Another novel pathway that originates from 5-HT in the DRN to somatostatin (SOM)-expressing and non-SOM interneurons in the central nucleus of the amygdala (CeA) in comorbid depressive symptoms (CDS) is identified. Inhibition of the 5-HT^DRN^→ SOM^CeA^ pathway produces depression-like behavior, and conversely, optogenetic activation of this pathway reduces depression-like behavior. These findings indicate that 5-HT^DRN^→ SOM^CeA^ might mediate some aspects of CDS (101). These studies imply altered 5-HT activity dynamics in the DRN after mPFC stimulation. Such altered neurotransmitter dynamics and network-based functional framework may help us understand depressive symptoms.

### Ventral tegmental area

As discussed in 2.1, VTA DA neurons have been extensively studied for their role in reward circuitry, emphasizing its possible link to depression (102).

Optogenetic induction of phasic firing in VTA DA neurons will rapidly induce a susceptible phenotype in mice undergoing subthreshold social defeat stress (SSDS) (45). Some studies investigate the effects of long-term up-regulation of VTA DA activity. This long-term change leads to antidepressive-like behavior in several experimental paradigms by chronically stimulating VTA DA neurons of unstressed and socially defeated mice (103, 104). The locus coeruleus (LC)→ VTA circuit also mediates homeostatic mechanisms in mesolimbic DA neurons. Stimulation of LC→ VTA neurons can promote a CSDS resilience-like phenotype and reverse hyperactivity of VTA→ NAc DA neurons (105).

Brain-derived neurotrophic factor (BDNF) plays numerous important functions in the brain, one of which is mediating the stress-induced depressive-like behavior *via* the VTA-NAC pathway. Optogenetic activation of VTA DA neurons projecting to NAc after SSDS paradigm reduces social interaction and increases BDNF levels in the NAc. Both of the effects disappear when BDNF signaling is blocked with the BDNF receptor antagonist tyrosine receptor kinase B (TrkB) or by using genetic BDNF knock-out mice (106). Additionally, the effects of DA VTA→ NAc stimulation during stress exposure can be blocked by infusing D1 receptor antagonist before social interaction testing in the CSDS model, however, not in SSDS. Optogenetic activation of the DA VTA→ NAc pathway after CSDS increases social avoidance without change of DA level in the NAc. Unexpectedly, the effects of social avoidance are blocked through TrkB or genetic BDNF knock-out, suggesting BDNF rather than DA signaling in the VTA→ NAc circuit is crucial for developing depressive symptoms after CSDS (107). DA neurons still work through other pathways to induce hypolocomotion performance. He et al. identified decreased projections from VTA to substantia nigra pars reticulata (SNr) with quantitative whole-brain mapping after CSDS protocol. Subsequent optogenetic stimulation of phasic firing of this projection could significantly increase the locomotor activity of mice. Their data suggests that the VTA-SNr dopaminergic projection involves in CSDS-induced hopolocomotion and provides a potential therapeutic target for MDD (108).

Ventral pallidum (VP) is a relayed nucleus that receives afferent from the NAc and transmits information to downstream targets such as the VTA and LHb (109). PV neurons in VP show increased activity in CSDS-susceptible mice, and silencing them can induce resilience to CSDS. Optogenetic silencing of VP PV neuronal projections to the VTA and LHb influences different depressive-like behavior, respectively. Inhibiting the former pathway exhibits alleviated behavioral despair with reduced immobility in TST, while the same manipulation of the LHb-projecting populations only improves social withdrawal levels in social interaction test (110). The lateral dorsal tegmentum (LDTg) is another essential component in reward responses and also an important input to the VTA (111). CSDS will cause hyperactivity of excitatory neurons projecting to the VTA. While the progression of depressive-like behavior is prevented by chemogenetically inhibiting LDTg neurons before daily defeat paradigm. This protective effect is then shown by inhibiting cholinergic neurons during CSDS, which implies that LDTg cholinergic inputs to the VTA drive maladaptation to social stress (112). Another possible circuit mediating the inhibitory influence is DRN→ VTA. Optogenetic stimulation of 5-HT input to the mesolimbic DA system combined with citalopram produces a synergistic decrease in responding to saccharin, a measure of motivated behavior. However, this inhibitory influence of DRN 5-HT neurons over responding for reward only produces in the VTA, which indicates that the effect is exerted through direct interaction with the mesolimbic DA system at the level of the VTA (113). Undoubtedly, the VTA has connections with many other brain regions that is waiting to be explored.

### Hippocampus

An earlier study has reported reduced hippocampal volume in patients with MDD, indicating smaller hippocampal volume might be a trait characteristic for MDD (114). Hippocampus can be divided into three functional segmentation in line with behavioral and anatomical studies including dorsal, intermediate and ventral, among which the ventral hippocampus (vHipp) regulates responses to stress, emotion and affect (115). Mice resilient to CSDS exposure show decreased glutamate release at vHipp-NAc synapses. Low-frequency stimulation to vHipp ChR2-infected mice decreases glutamatergic synaptic transmission at vHipp-NAc synapses. And these mice interact more time with a target mouse. Conversely, up-regulating glutamatergic synaptic transmission in NAc could increase stress susceptibility (116). Therapeutically, the vHipp-mPFC pathway enables an antidepressant response in Wistar-Kyoto (WKY) rats (117). Optogenetic activation of vHipp-mPFC can also mimic the antidepressant-like response to ketamine. Furthermore, optogenetic inactivation of this pathway during FST completely reverses ketamine's antidepressant response, indicating it may be involved in the rapid antidepressant effect of ketamine (118).

Upstream hippocampal circuitry, such as the entorhinal cortex (Ent), shows the potential for MDD treatment. Molecular targeting (Ent-specific knockdown of TRIP8b) and chemogenetic stimulation of Ent neurons induce antidepressive-like effects in mice. Ent stimulation-induced antidepressive-like behavior relies on the generation of new hippocampal neurons, indicating the controlled stimulation of the Ent-hippocampal pathway exerts anti-depressive effects *via* increased hippocampal neurogenesis (119). Hippocampal engrams represent ensembles of neurons with increased activity after memory formation. Zhang et al. examined social defeat-related hippocampal engrams in susceptible and resilient mice undergoing CSDS paradigm. TetTag mice were also used to label social defeat-related hippocampal ensembles by LacZ. Susceptible mice exhibited higher reactivating of social defeat-related LacZ-labeled cells in both the dorsal and ventral hippocampal CA1 regions compared with resilient and control groups. Activating or inactivating CA1 engram cells with optogenetic approaches enhanced and suppressed social avoidance, respectively. However, increased engram cells could not be found in the dentate gyrus. Taken together, all the evidence suggests that the susceptibility to CSDS is regulated by hippocampal CA1 engrams for negative memory (120). Other brain regions and neural circuits involved in depressive animal models are shown in Table 1.

**Table 1:** Other brain regions and neural circuits involved in depression.

**References**	**Neural circuit/brain region**	**Stimulation method**	**Test**	**Effects**
Vialou et al. (121)	PL-mPFC→ NAc PL-mPFC→ BLA	Optogenetics	SIT, EPM, SPT	Optogenetic stimulation of PrL glutamatergic projections to NAc reversed the social avoidance and increased sucrose preference, but did not affect anxiety-like behavior Optogenetic stimulation to BLA did not prevent the social avoidance and had no significant effect on sucrose preference Stimulation of BLA afferents increased time spent in the open arms of the EPM
Bagot et al. (116)	vHipp→ NAc mPFC→ NAc BLA→ NAc	Optogenetics	SIT, FST, OFT	Optogenetic activation of vHIP→ NAc after CSDS increased depressive-like behavior in SIT and FST, whereas depressing vHIP→ NAc glutamate synapses led to social avoidance Optogenetic stimulation of the glutamatergic mPFC→ NAc or AMY→ NAc synapses led to increased social interaction, while the stimulation of AMY→ NAc neurons decreased immobility in the FST. Anxiety-like behavior in the OFT was unaffected.
Teissier et al. (122)	DRN MRN	Chemogenetics	OFT, EPM, FST	Reduced DRN and increased MRN 5-HT activity correlated with increased anxiety and depressive-like behavior. DRN and MRN 5-HT neuron inhibition in naïve mice promoted and decreased FST immobility, respectively
Urban et al. (123)	DRN	Chemogenetics	DLET, OFT, FST	Acute activation of DRN 5-HT neurons induced anxiety-like responses in the LDET and OFT and reduced immobility in the FST. The antidepressive-like effect was observed in the FST as well as exploratory behavior in the home cage
Nishitani et al. (124)	DRN	Optogenetics	TST, OFT, EPM, FST	Acute activation of serotonergic neurons in the DRN increased active coping with inescapable stress in rats and mice, and acute inhibition of these neurons increased anxiety-like behaviors specifically in rats
Ohmura et al. (125)	DRN DRN→ VTA/SN	Optogenetics	EPM, FST, TST, 3-CSRTT	Optogenetic activation of 5-HT neurons in the DRN showed an antidepressive-like effect in FST and TST Activation of 5-HT terminals in the VTA/SN had an antidepressive-like effect in the FST Stimulation of BLA afferents increased time spent in the open arms of the EPM
Proulx et al. (126)	LHb→ RMTg	Optogenetics	FST, OFT, SPT	Activation of the LHb→ RMTg pathway reduced motivation to work for a reward in a progressive ratio operant task measured as maximal work performed to receive a sucrose solution Stimulation did not affect the preference for the sucrose solution over plain water in the SPT. Additionally, activation of the LHB→ RMTg pathway reduced motivation in the OFT
Warden et al. (98)	mPFC→ LHb	Optogenetics	FST	Optogenetic stimulation of local mPFC neurons did not have any net effect on the rats' behavioral state Optogenetic stimulation of mPFC axons in the LHb during FST led to a rapid decrease in mobility
Tchenio et al. (127)	LHb	Chemogenetics	Shuttle box test, SPT, TST	Limiting LHb neuronal activity through chemogenetics led to a remission of MS-driven behavioral phenotypes, with the treated mice showing a phenotype almost similar to the healthy control group
Anderson et al. (128)	PAC	Optogenetics	SPT, FST, OFT	Inhibiting PAC-Pdyn neurons induced transient depressive-like behavior, indicated by a decreased sucrose intake in the SPT and significantly more immobility during the FST
Cai et al. (129)	BLA→ CeA PBN→ CeA.	Optogenetics	OFT, FST, CPP, CPA	Acute optogenetic activation of the PBN→ CeA pathway was sufficient to drive anxiety- and depressive-like behavior in the OFT and FST. Acute activation of the BLA→ CeA pathway induced opposite behavioral effects. Simultaneous stimulation of both pathways failed to induce anxiety- or depressive-like behavior.
Ramirez et al. (130)	DG→ BLA→ NAc	Optogenetics	SPT, TST, NSFT	The BLA mediated a resilience effect induced by DG stimulation on a DG→ BLA→ NAc functional pathway, which was indicated by a decreased latency to feed, increased sucrose preference and increased amount of time struggling in the TST.

PL-mPFC, prelimbic medial prefrontal cortex; NAc, nucleus accumbens; BLA, basolateral amygdala; vHipp, ventral hippocampus; DRN, dorsal raphé nucleus; MRN, medial raphé nucleus; VTA, ventral tegmental area; SN, substantia nigra; LHb, lateral habenula; RMTg, rostromedial tegmental nucleus; PAC, periamygdaloid cortex; PBN parabrachial nucleus; CeA, central amygdala; DG, dentate gyrus; SIT, social interaction test; EPM, elevated plus maze; SPT, sucrose preference test; OFT, open field test; FST, forced swim test; DLET, dark-light emergence test; TST, tail suspension test; 3-CSRTT, three-choice serial reaction time task; CPP, conditioned place preference; CPA, conditioned place aversion; NSFT, novelty suppressed feeding test.

## Discussion

### The potential of optogenetics in clinical application

Advances in technology enables us to understand the mechanism of depression and also provides us with new perspectives and insights into its treatment. At present, almost 30% of patients with depression cannot achieve sustained remission with traditional drug treatment, which is defined as treatment-resistant MDD (TRD) (131, 132). But encouragingly, optogenetic approaches have contributed to the development and improvement of depression therapies.

Ketamine, a new fast-acting antidepressant, can exert an antidepressant effect within hours and produce sustained antidepressant actions for a week (133). A previous study has found that inactivating IL-mPFC can completely block the antidepressant effect of ketamine. And optogenetic stimulation of IL-mPFC produces rapid and long-lasting antidepressant effects. These results indicate that the therapeutic action of ketamine may take effect *via* IL-mPFC (134). Optogenetic disruption of the Drd1 expressing pyramidal cells in mPFC blocks the rapid antidepressant effects of ketamine. Furthermore, this antidepressant response measured by FST is completely blocked by photoinhibition of the mPFC-BLA pathway (135). Carreno et al. found activation of the vHipp-mPFC pathway was necessary and sufficient for the antidepressant response to ketamine and increase in TrkB receptor phosphorylation in the vHipp contributed to ketamine's sustained antidepressant response (118). As a newly discovered fast-acting antidepressant, ketamine does have much to be explored from the perspective of neural circuits utilizing optogenetics.

DBS is another physical therapy evolved to manage patients with TRD (136). The DBS electrodes implanted in the desired target of the brain are connected to a programmable pulse generator. Therapeutically, the generator stimulates neuronal populations and elements to influence local, regional, and up-and downstream projections. However, the identification of the networks and their constituent elements is challenging, and the nature of the network disturbances and their precise location and dynamic properties are also incompletely understood (137).

In some clinical trials, TRD patients receiving DBS treatment show great differences in response rate during the follow-up (138, 139). The reason for these discrepancies may lie in the individual differences in the structure and function of neural circuitry and network. In Papp's study, they used WKY mice, a model resistant to antidepressant treatment, to compare the effects of DBS and optogenetic stimulation on animals' behavior. DBS had minor non-specific effects on exploration and locomotor activity in the novel object recognition (NOR) test. Although optogenetic stimulation of the vHipp-mPFC pathway did not reverse the effect of CMS on impaired NOR, it increased exploratory behavior and locomotor activity in the NOR test. A likely explanation is that DBS excites both intrinsic cells and afferents to PFC in the infected region, whereas optogenetic stimulation of mPFC excites only intrinsic cells (140). We have yielded greater progress by combing DBS with optogenetics in neurological and psychiatric diseases such as Parkinson's Disease and addiction (141–143). But currently, this field in animal models about depression is still blank. Considering the advantages of optogenetics in precise circuit regulation, future researches can seek specific circuits that change in DBS, ultimately reaching the goal of accurate treatment and less damage to the brain.

### Methodical limitations

The current generation of optogenetic tools has been adapted to extensive questions in neuroscience and has also been optimized for stronger expression, currents and spectral shifts. Despite its potential and convenience, optogenetics has limitations and challenges to be addressed before further development and use. The main problems include infection, bleeding, and injury to the brain tissue caused by fiber insertion; restriction on animal's activity and natural behavior (16); and heat accumulation during the illumination (144, 145). Fortunately, the appearance of genomics-improved opsin, tapered optical fiber, uLED and wireless technology has improved these problems to a large extent (29–31, 146). We believe ongoing advances in optogenetic toolbox will yield tools that expand the optical control of biochemistry even further.

When it comes to depressive-like behaviors, we mainly summarize recent optogenetic findings in anhedonia, appetite change, and social avoidance. Besides, sleep disturbance is also an unignorable symptom that occurs in up to 70% of MDD patients (147). Although a slew of researches have investigated the relationship between sleep and neural circuits with optogenetics, only a small portion of them consider this issue in the context of depression, which may highlight the importance of considering sleep problems and other symptoms in MDD patients. As previously discussed, symptoms such as the feeling of worthlessness, self-guilt and suicidal ideation can neither be observed in animal models nor be measured quantitatively by behavioral tests, further restricting our steps toward the mechanisms of these depressive-like behaviors. Therefore, we urgently need new tests to detect depressive-like behaviors that have never been measured before to advance our research to new heights.

## Future perspective and conclusion

To summarize, our review provide evidence for a complicated neural circuit that has been hypothesized to casually underlie depression. But we had one limitation that we only discussed brain regions and neural circuits that have been studied most in animal models. Due to the complexity of neurocircuitry in depression, the identified circuits in this review are just a subset dissected from the whole brain. There is also emerging evidence to explain the role of regions previously unknown, like medial dorsal thalamus (MDT) and anteroventral subdivision of the bed nuclei of the stria terminalis (avBST) (148, 149). More attention should be attached to these regions, aiming to construct an all-around understanding of our brain networks. Besides, some studies have found stimulation of sub-circuits may produce different effects, reminding us that we should not only concern about the overall effects of neural pathways, but also notice sub-regions/circuits with optogenetics in the future.

In conclusion, this paper reviews the recent progress of optogenetic applications in exploring the etiology of depressive-like behaviors at the circuit level. This unique neuroscience technique has increased our understanding of this complicated psychiatric disorder, and soon it may be useful in improving and optimizing clinical therapy for depression.

## Author contributions

SL and GW participated in the design of the study, carried out the data collection, drafted the manuscript, and edited the manuscript. YD, YXia, and YXie helped with the analysis. LX advised on the article ideas and helped to draft the manuscript. All authors read, edited, and approved the final manuscript.

## Funding

This study was supported by the National Natural Science Foundation of China (Nos. 81871072 and 82071523) and the Medical Science Advancement Program of Wuhan University (No. TFLC2018001). Design of this study was supported by the Key Research and Development Program of Hubei Province (2020BCA064).

## Conflict of interest

The authors declare that the research was conducted in the absence of any commercial or financial relationships that could be construed as a potential conflict of interest.

## Publisher's note

All claims expressed in this article are solely those of the authors and do not necessarily represent those of their affiliated organizations, or those of the publisher, the editors and the reviewers. Any product that may be evaluated in this article, or claim that may be made by its manufacturer, is not guaranteed or endorsed by the publisher.
